# Formalizing our commitment to code sharing

**DOI:** 10.1371/journal.pbio.3003678

**Published:** 2026-02-26

**Authors:** Nonia Pariente, Lauren Cadwallader, on behalf of the PLOS Biology Staff Editors

**Affiliations:** 1 Public Library of Science, San Francisco, California, United States of America and Cambridge, United Kingdom

## Abstract

In support of open science, PLOS Biology routinely asks authors to openly share their research code before publication. We are now formalizing this practice with a mandatory code sharing policy and clarifying what we talk about when we talk about code.

An increasing amount of modern life sciences research relies on computational biology. Whether you are developing a model to help answer your research question, writing code for data cleaning, analysis, or visualization, or using small scripts to run off-the-shelf packages, research code abounds. Chances are, there is an element of code in your articles, even if you have not generated it yourself, are not familiar with best practices in annotating, sharing, or licensing it, and may not even recognize it as such.

In addition to the narrative manuscript and the data associated with it, code is increasingly necessary to build on research and continue to move science forward. It can give important context to fully understand how data was analyzed and allows others to perform similar analyses with their own dataset(s). *PLOS Biology* has always been committed to promoting open science practices. As part of these efforts, we have asked authors of all of our accepted articles to share their code for the past 3 years, and have experienced little to no pushback. Thus, at the start of this year, we formalized our commitment to the sharing of research code by implementing a new mandatory code-sharing policy for all articles.

According to our latest PLOS Open Science Indicator dataset, code sharing has been increasing at *PLOS Biology* year-on-year. However, the data suggest that a gap remains between those articles producing code and those sharing it. There is clearly room for improvement, and we think a barrier to higher sharing rates is an incomplete understanding of what needs to be shared. Of all the checks that we do before accepting a study for publication, such as ethics compliance, conflicts of interest, adherence to our data sharing policy, or data deposition in relevant databases, we find that our authors are least familiar with what is required with respect to code.

Working on the policy text in consultation with community experts has allowed us to better define the specific requirements of the policy and prepare our editorial team to help authors navigate what we mean by novel code generation (Box 1). To help bring clarity, our policy is explicit, and we have developed detailed accompanying code-sharing guidance. We are happy to work with and support our authors on this important step towards a more open science practice.

**Box 1. Navigating the *PLOS Biology* mandatory code-sharing policy**

**What?**

All author-generated code required to reproduce the findings, analysis, and visualization reported in the manuscript, as well as related documentation. This includes custom code executed within commercial software packages (e.g., Matlab, SPSS, MS Office software)

For example, you are required to share the following code:

- Novel, in-house, or custom code/scripts written for data processing or analysis.
- New computational models and software packages.
- Existing software/packages/models that use a coding language (e.g., Python, R, MATLAB), which are modified by the authors and used to process or analyze data.
- New code written for simulations.

**Where?**

Code can be deposited in a permanent, public repository that issues persistent identifiers. Where code has been shared in a non-permanent repository, such as Github or Bitbucket, a snapshot of the code should be archived in a permanent, public repository, for example, Zenodo, Code Ocean, or the Software Heritage archive. A statement about where and how your code can be accessed must be included in the Data Availability Statement in your manuscript. Please link to both the archived snapshot and live versions of the code, as they serve different purposes and both will be useful for readers.

**When?**

Code will need to be shared before publication, and we will request that it is made publicly available during our pre-acceptance checks. Editors and reviewers may request to see your code, especially if it is central to the claims of the study. Some repositories, such as Github, figshare, or Code Ocean, will allow you to share your work with others privately, and we ask that you include these links in your cover letter at submission.

**Why?**

Sharing code supports open, transparent research that enables other researchers to assess, reuse, and reproduce published work.

**How?**

Please include:

- A README file describing the code in an accessible manner, the environment in which it should be run, and any existing dependencies. You should also state the version of the code you are sharing and a link to the live code so users can check for any updates.
- A LICENSE.txt file that specifies the copyright holder and terms under which others can use, modify, and share your code.

Upon publication, any code generated that relates to the results described in the article (including their analysis or visualization) must be shared publicly and in perpetuity, unless you have an acceptable exemption. Code does not need to be shared before publication, but should be available to editors and reviewers at the time of submission and throughout the editorial process (Box 1). We do not require that you apply any specific license to your code, only that a license is indicated, but we strongly recommend a license that supports open, reproducible research, if it is compatible with institutional and funder policies. We also encourage authors to use complete open-source solutions where possible. If you are reusing existing code without modification, we encourage you to share it where possible or to cite the code used in the references section. We are not changing the way we approach peer review, and authors remain responsible for the quality of the code and accompanying documentation, although where code is a crucial part of the manuscript, reviewers are (as currently) likely to assess it during peer review.

Code deposition should follow best practice to enable access in a useful and reusable way. It should be time-stamped and associated with a persistent identifier, such as a digital object identifier (DOI) or software hash identifier (SWHID). A persistent identifier provides a permanent reference to the version of the code that was used in the article, even if this is subsequently changed, enabling accurate citation and transparency. Linking to the live version of the code as well will allow readers to see any further development carried out since publication.

We understand that there may be valid reasons for needing an exemption from code sharing such as ethical restrictions, legal limitations (e.g., contractual obligations), or dual-use concerns. In such cases, please note your request for an exemption in the cover letter for editorial assessment. We recommend using a controlled access deposit to a repository to facilitate access requests, or identifying the contacts to whom requests should be submitted in the Data Availability Statement. Where a study’s data is subject to sensitivity concerns but the code is not, the code can be provided alongside simulated datasets.

We have introduced this policy not only because we believe open science accelerates discovery, but also because our experience over the past three years indicates that our community is ready for and supports code sharing. For many authors, this policy is a formalization of an existing practice they have already encountered at the journal. We encourage any authors who are new to code sharing to reach out to our editorial team, who will be happy to guide you through the process. Many institutions also offer support for open science, and there are guides to code-sharing practices, including recommendations for those who are new to this practice. Stay tuned for reports on our progress and please do get in touch with any feedback. Thank you for helping support open science practice—together we are a catalyst for better.

